# The postpartum effect of maternal diabetes on the circulating levels of sirtuins and superoxide dismutase

**DOI:** 10.1002/2211-5463.12370

**Published:** 2018-01-08

**Authors:** Samar Sultan, Nada Alzahrani, Kalthoom Al‐Sakkaf

**Affiliations:** ^1^ Faculty of Applied Medical Sciences King Abdulaziz University Jeddah Saudi Arabia

**Keywords:** gestational diabetes, oxidative stress, postpartum, SIRT1, SIRT3, SOD2

## Abstract

Gestational diabetes mellitus (GDM) is a glucose intolerance disorder which occurs during pregnancy as a result of insulin insensitivity; it usually disappears after delivery. However, some women with GDM can develop type 2 diabetes (T2D) after delivery, and the mechanisms by which this occurs remain unknown. This study compared the levels of sirtuins (NAD‐dependent deacetylases) and antioxidative enzymes in postpartum women with previous GDM (pGDM) or T2D and in postpartum women with a previous healthy pregnancy (controls). Women with pGDM showed upregulated levels of sirtuin 1 (SIRT1) mRNA and protein, with reduced expression levels of sirtuin 3 (*SIRT3*) and superoxide dismutase 2 (*SOD2*), relative to the controls. Women with T2D similarly showed a lower level of SIRT3 mRNA than the controls. Lipid peroxidation (malondialdehyde) was higher in women with pGDM than in the controls. These data show that in women with pGDM, the reduced level of *SIRT3* may play a role in the reduced *SOD2* level, possibly leading to oxidative stress, which, in turn, upregulates the level of *SIRT1*. These results might confer the risk of future diabetes development in women with pGDM, as a similar reduction in *SIRT3* was found in women with T2D.

**Abbreviations**
BMI: body mass index
F.Ins: fasting insulin
FPG: fasting plasma glucose
GDM: gestational diabetes mellitus
HbA1c: glycosylated hemoglobin
MDA: malondialdehyde
NFkB: nuclear factor kappa B
PARP: poly (ADP‐ribose) polymerase
pGDM: previous GDM
ROS: reactive oxygen species
RPG: random plasma glucose
SIRT: sirtuin
SOD: superoxide dismutase
T2D: type 2 diabetes

Diabetes mellitus, a metabolic disease characterized by chronic hyperglycemia, with disturbances in the metabolism of carbohydrates, proteins, and fats, is due to defects in insulin action or insulin secretion or both. The number of people with diabetes stood at around 171 million worldwide in 2000, and this is predicted to increase to about 366 million cases by 2030 1. During pregnancy, women can develop gestational diabetes mellitus (GDM) as a result of insulin insensitivity, but this usually disappears after delivery. However, some of these women with previous GDM (pGDM) develop type 2 diabetes (T2D) later in life, the reason for which is still unknown. Many studies have reported that the risk of T2D is increased for patients with GDM 2, 3, 4. It has been shown that pregnant women with GDM exhibit a hyperglycemia‐induced increase in circulating oxidative stress and reduction in the antioxidative enzymes 5, 6, the mechanisms of which are not yet fully understood. Thus, increased oxidative stress may result in adverse effects to both the mother and fetus, including the development of T2D and cardiovascular diseases later in life.

Sirtuins are NAD‐dependent deacetylases that have a wide spectrum of metabolic and stress‐tolerance properties. Among them are the well‐characterized antioxidative and cytoprotective sirtuin 1 (SIRT1) and sirtuin 3 (SIRT3). Mammalian SIRT1 can activate or suppress the expression of target genes involved in aging, cellular metabolism, and stress response by deacetylating substrates, including histones, coregulators, and transcription factors 7. In addition, it has recently been shown that SIRT1 modulates apoptosis, the cell cycle, energy homeostasis, and reactive oxygen species (ROS) levels 8, 9. However, whether an alteration in the level of circulating SIRT1 in postpartum women with pGDM is related to oxidative stress has not been investigated. High glucose (30 mm) reduces SIRT1 at the mRNA and protein levels and hence its activity 10. A persistent or metabolic memory of hyperglycemic stress has been reported to be abrogated by SIRT1 protein through the LKB1/AMPK pathway 10. In a primary bovine retinal capillary endothelial cell culture model exposed to high glucose (30 mm) for 1 week followed by normal glucose conditions for 2 weeks, the cells showed persistent gene and protein expression of the inflammatory and apoptotic nuclear factor kappa B (NFkB), poly (ADP‐ribose) polymerase (PARP), and Bcl‐2‐associated X protein (Bax), which are involved in diabetic vascular complications 10. In this model, the SIRT1 level remained low even after glucose normalization for 2 weeks, suggesting endothelial programming by the exposure to hyperglycemia; however, the exact mechanisms involved are unknown. The authors of this study treated these cells with either metformin (a drug prescribed for T2D) or the SIRT1 activator resveratrol, both of which increased the expression of SIRT1 while reducing the expression levels of NFkB, Bax, and PARP, suggesting that the ROS‐induced PARP activation under the hyperglycemic condition was at least partially reduced by the expression of SIRT1.

Recent studies have reported that SIRT3 regulates high‐glucose‐induced oxidative stress through the deacetylation and activation of superoxide dismutase 2 (SOD2) 11, 12, and the activity of this enzyme is inhibited when the *SIRT3* gene is deleted 13. *SIRT3* is the downstream target gene of peroxisome proliferator‐activated receptor‐gamma coactivator‐1 alpha and regulates mitochondrial biogenesis 14. In addition, the study showed that *SIRT3* gene knockdown increased apoptosis and cellular ROS in pancreatic islet beta cells isolated from patients with T2D 15. Malondialdehyde (MDA), the product of lipid peroxidation, is generated by the ROS‐induced degradation of phospholipids under pathological conditions, such as diabetes mellitus 16, 17.

The aim of this study was to test the alterations in the levels of circulating sirtuins and SOD in postpartum women with pGDM or T2D in relation to hyperglycemia‐induced oxidative stress.

## Materials and methods

### Subjects

For this study, 15 pregnant women with GDM (GDM group), 13 pregnant women without diabetes (control group), and five pregnant women with T2D (T2D group) were recruited from the Obstetrics and Gynecology Department of the King Abdulaziz University Hospital, Jeddah, Saudi Arabia. All the donors were informed about the objectives of the research, and informed consent was obtained from each study participant prior to sample collection. This study was approved by the Ethics Committee of the King Abdulaziz University Hospital. Exclusion criteria for the control group were as follows: subjects on medication or who have a family history of diabetes and/or high blood pressure and/or are smokers. Inclusion criteria for the GDM group were as follows: a history of GDM in the second or third trimester of gestation, and absence of infection, hypertension, and/or any other concomitant systemic diseases. Subjects enlisted had a previous fasting plasma glucose (FPG) concentration of > 5.8 mmol·L^−1^ and postload plasma glucose concentration at 2 h of > 11.1 mmol·L^−1^ for a 75 g oral glucose tolerance test performed during pregnancy 18. For glucose control, all women in the GDM group were on dietician‐recommended diets, except for one woman who was on insulin. Subjects with a FPG concentration of > 7 mmol·L^−1^ were considered as type 2 diabetic according to the American Diabetes Association 19. All the patients with T2D were on insulin along with other medications, such as metformin and novorapid. Height, weight, and body mass index measurements were recorded at approximately 12 weeks of pregnancy. For all experiments, including real‐time PCR, ELISA, and MDA tests, samples were obtained from a minimum of five to seven subjects from each study group.

### Biochemical measurements

A venous blood sample was collected for glycosylated hemoglobin (HbA1c) and random plasma glucose (RPG) analyses on the day of delivery. The level of circulating HbA1c, an indicator of blood glucose control, was measured with a Hitachi 911 autoanalyzer (Hitachi Co. Ltd., Tokyo, Japan). Normal values of HbA1c as reported previously 20 ranged from 4.0% to 6.0%.

At 1 day postpartum, blood samples were collected again from all three study groups for FPG, fasting insulin, ELISA, and lipid peroxidation (MDA) analyses. The 5 mL of fasting venous blood sample obtained was centrifuged at 3000 ***g*** for 15 min, and the plasma samples were stored at −80 °C until assayed. The fasting blood glucose concentration and insulin levels were measured by an automated enzymatic method (Roche Diagnostics GmbH, Mannheim, Germany). Another 3 mL of blood was collected directly into plain tubes for the ELISA and MDA experiments. The obtained serum samples were stored at −80 °C until processing. Serum concentrations of SIRT1 and MDA were measured with an ELISA system (SIRT1 ELISA; Cusabio Co., Suffolk, UK) and a colorimetric assay kit (Sigma‐Aldrich Co. Ltd., Irvine, UK), respectively, following the manufacturers’ instructions.

### Real‐time PCR

For the genetic test, 2.5 mL of blood was collected into PAXgene blood RNA tubes at 1 day postpartum and stored at −80 °C until processing. Total cellular RNA was isolated from the samples with the PAXgene blood RNA kit (Qiagen, Manchester, UK) as per the manufacturer's instructions. The RNA concentration and purity were assessed spectrophotometrically, and the integrity was evaluated using an Agilent 2100 Bioanalyzer (Agilent, Edinburgh, UK). cDNA was synthesized from 1 μg RNA using an ImProm‐II Reverse Transcription System kit (Promega, Southampton, UK) following the manufacturer's protocols. The SIRT1 primer was designed using the Primer3Primer design tool, whereas the primers for β‐actin (reference gene), SIRT3, and SOD2 were taken from earlier studies 14, 21, 22. The sequences of all the primers are summarized in Table 1. β‐Actin mRNA was used as an internal control for normalization of the mRNA levels among the samples. Amplifications were performed in duplicate, using a QuantiTect SYBR Green PCR kit (Qiagen), in the iCycler iQ Real‐time PCR Detection System (Applied Biosystems, Cheshire, UK), according to the methods detailed in the manufacturer's instruction manual. After 10‐min denaturing at 95 °C, 40 cycles of amplification were carried out (denaturing at 95 °C for 15 s, annealing at 63 °C for 10 s, and extension at 72 °C for 20 s). Relative expression quantification was carried out using rest 2009 software version 2.0.13 23.

**Table 1:** Primers used for RT‐PCR

Primers	Sequences
SIRT3	Forward: 5′‐CGGCTCTACACGCAGAACATC‐3′
	Reverse: 5′‐CAGCGGCTCCCCAAAGAACAC‐3′
SOD2	Forward: 5′‐CGACCTGCCCTACGACTAC‐3′
	Reverse: 5′‐TGACCACCACCATTGAACTTC‐3′
β‐Actin	Forward: 5′‐TCATCACCATTGGCAATGAG‐3′
	Reverse: 5′‐CACTGTGTTGGCGTACAGGT‐3′
SIRT1	Forward: 5′‐CCAGCCATCTCTCTGTCACA‐3′
	Reverse: 5′‐TGGTTTCATGATAGCAAGCGG‐3′

### Statistical analysis

Student's *t*‐test was applied for unpaired data. Results are represented as mean ± SEM of a minimum of four independent values, and differences among means were considered statistically significant when *P *<* *0.05.

## Results

**Figure 1:**
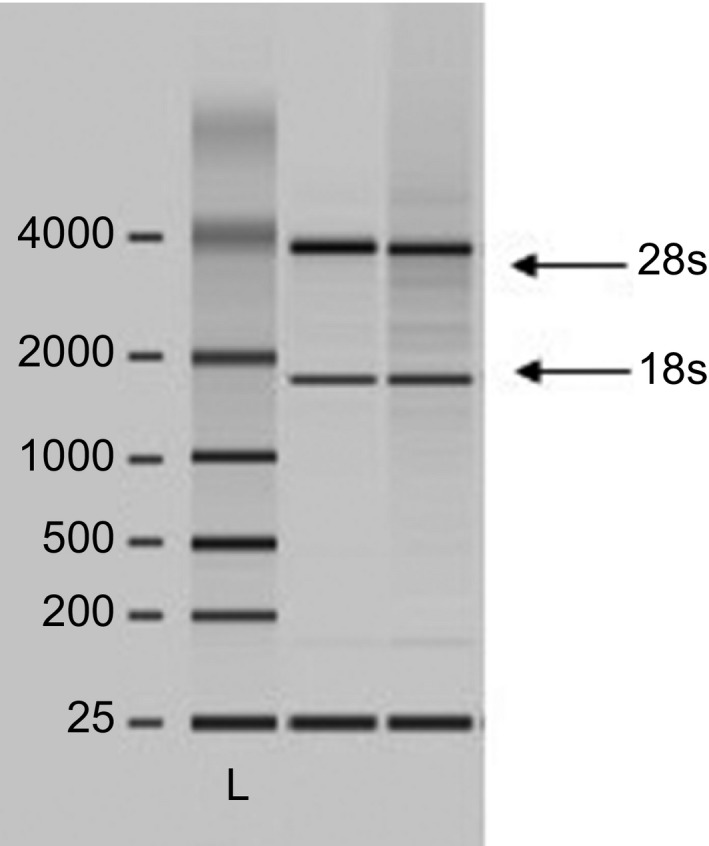
A representative image of the total RNA isolated from whole blood collected in PAX tube and evaluated using the Agilent 2100 Bioanalyzer. The gel electrophoresis showed the ratio of the ribosomal RNA bands of 2 : 1 for 28S and 18S, respectively. L, ladder.

**Figure 2:**
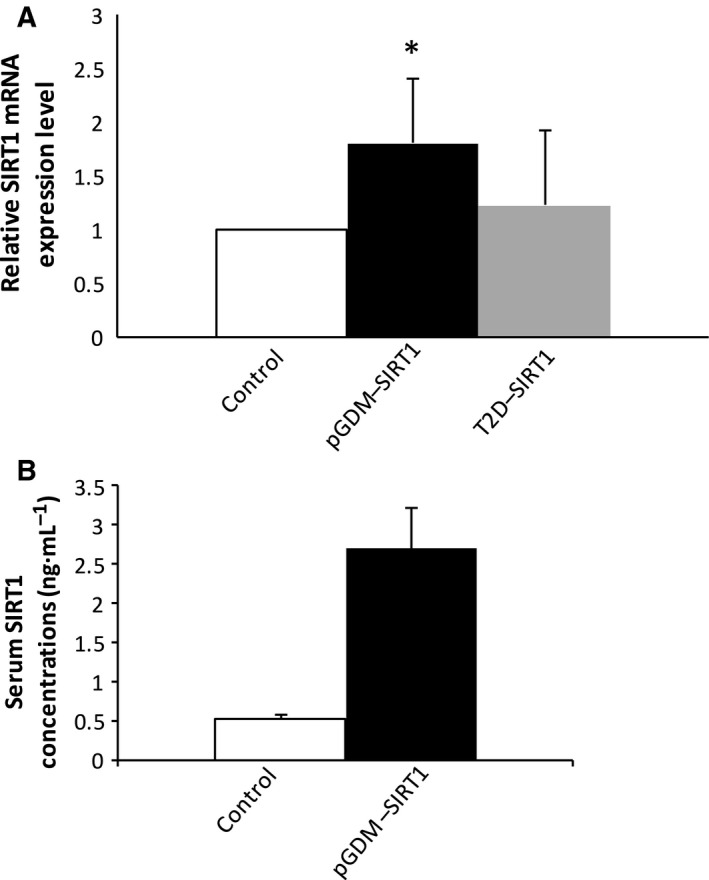
Expression levels of SIRT1 in patients with pGDM and T2D. The evaluation of the expression levels of SIRT1 and β‐actin is shown for control, pGDM, and T2D (A). SIRT1 expression was measured by real‐time PCR. Secretion of SIRT1 was measured by ELISA in control and pGDM (B). Data are presented as means ± SEM of five to six different donors (*n* = 5–6). *P < 0.05 vs controls.

**Figure 3:**
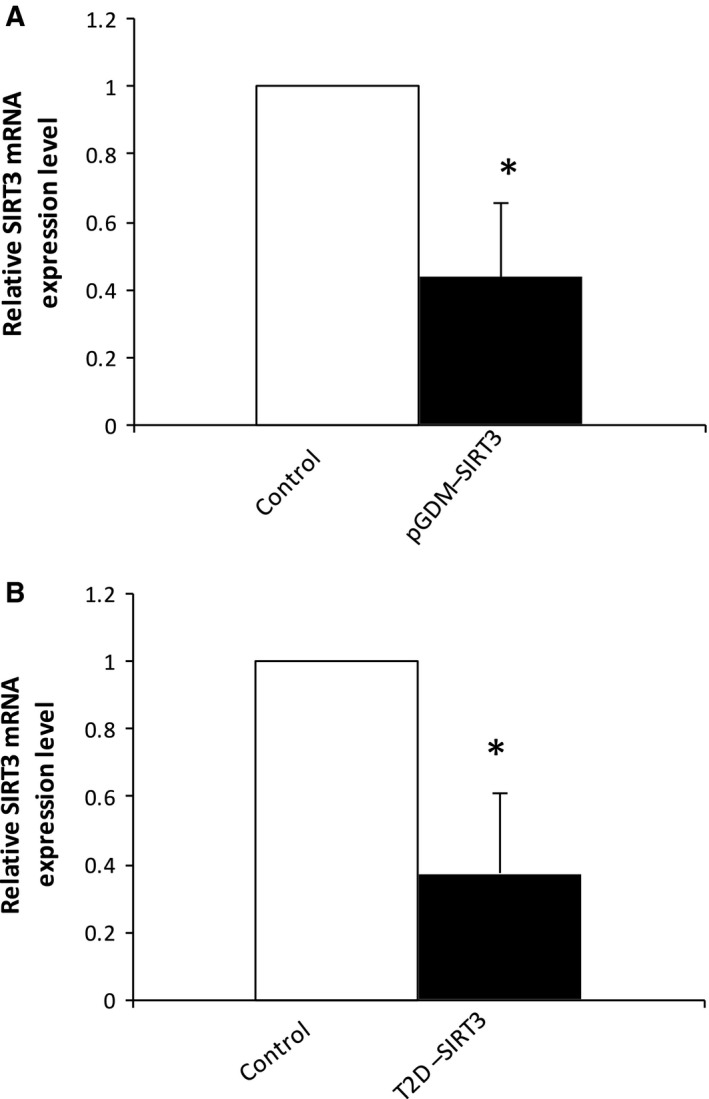
Expression levels of SIRT3 in patients with pGDM and T2D. The evaluation of the expression levels of SIRT3 and β‐actin is shown for both control and pGDM (A). The evaluation of the expression levels of SIRT3 and β‐actin is shown for both control and T2D (B). SIRT1 expression was measured by real‐time PCR. Data are presented as means ± SEM of five to seven different donors (*n* = 5–7). *P < 0.05 vs controls.

**Figure 4:**
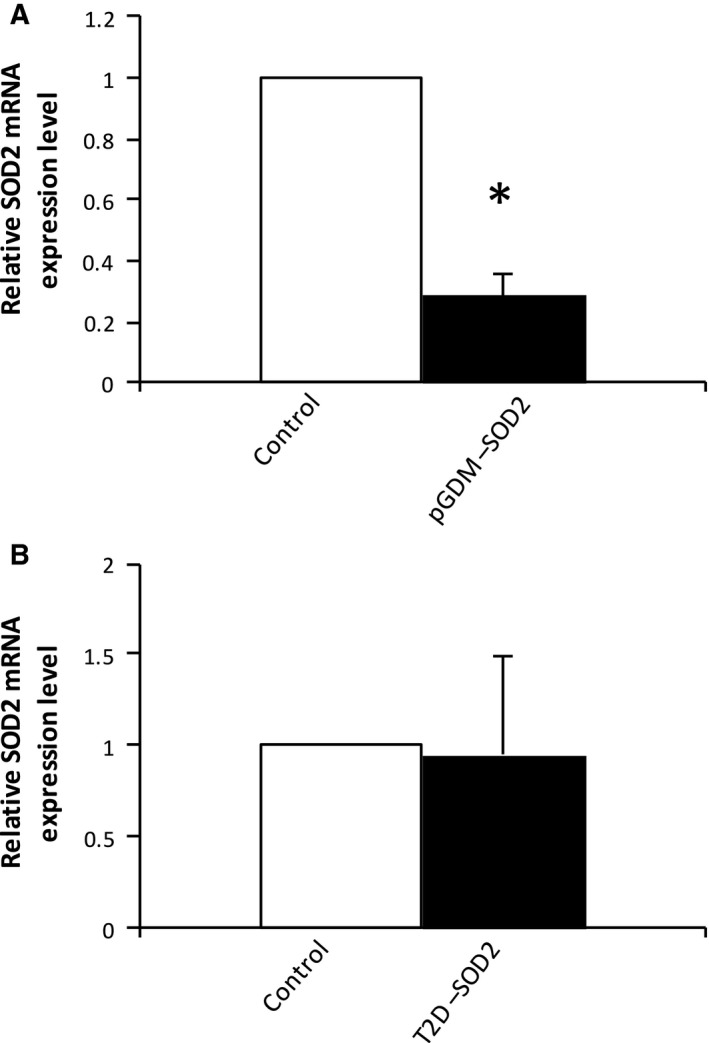
Expression levels of SOD2 in patients with pGDM and T2D. The evaluation of the expression levels of SOD2 and β‐actin is shown for stated groups in (A) and (B). SOD2 expression was measured by real‐time PCR. Data are presented as means ± SEM of five to seven different donors (*n* = 5–7). *P < 0.05 vs controls.

The clinical characteristics of the subjects in this study are summarized in Table 2. The women's age and HbA1c and RPG levels were significantly higher in the GDM group than in the healthy control group. There were no significant differences in other parameters between the two groups. However, the women in the T2D group had higher random glucose levels before delivery and higher FPG levels at 1 day postpartum relative to the levels in the control group. As shown in Table 2, serum MDA, a biomarker of oxidative stress, was nonsignificantly higher in the GDM group than in the control group postpartum (*P *=* *0.1, *n* = 10). However, there were no significant changes in MDA levels in the T2D group relative to the control group (Table 2). To investigate the effect of GDM on the postpartum mRNA expression levels of sirtuins and antioxidative enzymes, RNA were isolated from whole blood derived from the women in the three groups at 1 day postpartum. As depicted in Fig. 1, the 28S and 18S ribosomal RNA bands were in a ratio of 2 : 1, which confirmed that these RNA were of a good enough quality to be used in the real‐time PCR. As determined by real‐time PCR, the SIRT1 mRNA level was significantly higher (*P *=* *0.027, *n* = 6) in the patients with a history of GDM than in the healthy controls when compared with the housekeeping gene *β‐actin* (Fig. 2A). The serum SIRT1 protein concentration was also increased in an almost significant manner (*P *=* *0.055, *n* = 6) (Fig. 2B). On the other hand, there was a nonsignificant increase in the SIRT1 mRNA level in the T2D group relative to the control group (*P = *0.74, *n* = 5) (Fig. 2A). Interestingly, the patients with a history of GDM exhibited a significantly lower level of SIRT3 mRNA (*P *=* *0.044, *n* = 6–7) (Fig. 3A) than did the control group. A similar finding was also observed between the T2D and control groups (*P *=* *0.037, *n* = 5) (Fig. 3B). To examine the expression level of antioxidative enzymes in patients with pGDM, we analyzed the *SOD2* expression level by real‐time PCR. As shown in Fig. 4A, there was a significantly lower level of SOD2 mRNA in the GDM group than in the control group postpartum (*P *=* *0.046, *n* = 7). However, the expression level of SOD2 mRNA did not differ between the T2D and control groups (*P *=* *0.85, *n* = 5) (Fig. 4B).

**Table 2:** Clinical characteristics of subjects participating in the study. F.Ins, fasting insulin; BMI, body mass index. Data are expressed as mean ± SEM (range). Bold numbers indicate significant data

Status of mothers	Controls (*n* = 13)	GDM (*n* = 15)	T2D (*n* = 5)	*P* (GDM vs controls/T2D vs controls)
Age (years)	26 ± 0.3	30 ± 0.4	32 ± 1	**0.03**/0.08
Weight^a^ (kg)	69 ± 12	76 ± 1.3	74 ± 2.6	0.16/0.12
Height (cm)	157.8 ± 0.01	158 ± 0.06	157 ± 0.01	0.44/0.42
BMI	27.7 ± 0.4	30 ± 0.5	30 ± 1	0.14/0.12
HbA1c (%)	5.4 ± 0.0 (*n* = 6)	6.1 ± 0.04 (*n* = 11)	6 ± 0.1	**0.003**/0.055
RPG (mm)	4.2 ± 0.04 (*n* = 11)	5.15 ± 0.2 (*n* = 12)	5.2 ± 0.1 (*n* = 4)	**0.046**/**0.005**
Postpartum FPG (mm)	4.9 ± 0.07 (*n* = 12)	5.17 ± 0.11 (*n* = 11)	5.65 ± 0.16 (*n* = 4)	0.26/**0.049**
Postpartum F.Ins (mIU·L^−1^)	21 ± 2.5 (*n* = 7)	19.8 ± 1.5 (*n* = 9)	31.8 ± 4 (*n* = 4)	0.46/0.11
MDA (nmol·L^−1^)	0.098 ± 0.0 (*n* = 10)	0.143 ± 0.01 (*n* = 10)	0.082 ± 0.002	0.192/0.063

^a^At 12 weeks of pregnancy.

## Discussion

Women with GDM can develop T2D after delivery, the mechanisms of which are unclear.

In this study, we found that the expression of circulating SIRT1 was increased at the mRNA and protein levels in women with pGDM, whereas the expression levels of *SIRT3* and *SOD2* were decreased; this may be accompanied by pathological damage. On the other hand, women with T2D showed significant change in *SIRT3* level only, supporting the notion that the glycemic programming of gene expression in women with pGDM could result from a recent exposure to hyperglycemia induced by GDM. It also shows that these altered genes can potentially serve as diabetes risk factors, although this needs to be further investigated in a large cohort study. SIRT1 localized in the nucleus is implicated in mitochondrial biogenesis 24 and regulates glucose metabolism 25 through the enhancement of insulin sensitivity 26. In the present study, we found a significant increase in its mRNA expression and an almost significant increase in its protein level. It is noted that we could verify only the protein expression of highly expressed genes, as the detection of protein from lowly expressed genes was below the limit of detection of the ELISA. Ambra *et al*. 27 reported an upregulation in *SIRT1* expression in GDM patient‐derived human umbilical vein endothelial cells cultured under normal glucose conditions. It can be inferred from that study that the increased *SIRT1* expression postdelivery seen in our model may be due to a recent history of GDM. The underlying mechanism remains to be elucidated. Although not significant, we also observed a slight increase in the expression of *SIRT1* in the women with T2D. Contradictory results were reported by Balestrieri *et al*. 28, who showed a reduction in SIRT1 levels in endothelial progenitor cells isolated from patients with T2D. This could be explained by differences in the cell types, conditions, and experiments applied by the different studies.

High level of MDA has been reported in patients with T2D as a result of hyperglycemia‐induced ROS production and lipid peroxidation 16, 17. In the current study, we found a nonsignificant increase in MDA in the GDM group relative to that in the control group (Table 2). An *in vivo* study showed that SIRT1 expression increased to fourfold in response to oxidative stress (paraquat injection) in adult mouse and monkey hearts 29. Furthermore, it has been shown that whereas mild ROS levels trigger SIRT1 expression 30, a harsh exposure to ROS may reduce SIRT1 expression and activity 31. Thus, the increase in *SIRT1* expression in women with pGDM could be also due to the mild oxidative stress observed in the present study.

SIRT3, located in mitochondria 32, is another member of the sirtuin family that regulates energy homeostasis 33 and oxidative metabolism 11. Mitochondrial SIRT3 removes excess cellular ROS by removing acetyl groups from target proteins such as SOD2, thereby activating these antioxidative enzymes under different pathological and physiological conditions 34. As mentioned in our first postpartum analysis of women with GDM and T2D, the level of *SIRT3* expression was decreased in both groups, which can lead to altered mitochondrial function through increased ROS generation and oxidative stress development. Reduced SIRT3 expression has been verified in other models of T2D 15 and GDM 35. It has been shown that *SIRT3*‐knockout mice exhibit metabolic changes, including increased oxidative stress 13. In addition, the study showed that *SIRT3* deletion plays a role in impairment of beta‐cell function in T2D through accelerated development of the metabolic syndrome 36. In fact, evidence of increased oxidative stress has been reported in maternal cord blood 37 and endothelial cells derived from women with GDM 38 and has been linked to fetal programming, which in turn increases the development of T2D in the offspring in later life 39, 40. ROS plays an important role in the development of T2D 41.

Antioxidative enzymes, such as SOD2, glutathione peroxidase, and catalase, exert defense mechanisms to alleviate the excess ROS and prevent oxidative stress 42. In this study, the expression of *SOD2*, the key superoxide scavenger, was markedly decreased in women with pGDM relative to that in the healthy women, which was in line with studies that have documented reduced antioxidative enzyme levels in diabetic animal models 43, 44, individuals with T2D 45, and women with pGDM 46. However, the mechanisms involved need to be explored. Under normal conditions, SIRT3 enhances the expression of SOD2 mRNA and causes deacetylation of its protein in response to oxidative stress 34. Therefore, it is tempting to speculate that the low expression of *SIRT3* may account for the low *SOD2* expression in our model. However, the level of SOD2 mRNA did not differ between the T2D and control groups. Nonetheless, *SIRT3* expression was reduced in the T2D group, which might be implicated in the pathogenesis of T2D.

We recognize that this study is limited by the low number of study subjects; nevertheless, the statistical significance values obtained strongly confirm the validity of the results. However, further study with a larger number of cases is recommended to explore the mechanisms behind the results observed in the current study. There were no significant differences in characteristics of the subjects except for the significant increases in HbA1c and random glucose levels and higher age of the women with pGDM. In addition, elevated random glucose and postpartum fasting glucose levels were noted in the women with T2D.

The results of the current study showed alterations in the levels of *SIRT1*,* SIRT3*, and *SOD2* in women with pGDM. These alterations can confer an increased risk for the development of T2D in women with pGDM as a similar reduction in *SIRT3* was also found in women with T2D. Therefore, we believe that measures to enhance SIRT3 expression and its activity would be useful to protect women against the development of diabetes.

## Author contributions

SS conceived and designed the project. SS and NA acquired the data. SS, NA and KS analyzed and interpreted the data. SS wrote the paper.

## References

[1] Rathmann W and Giani G (2004) Global prevalence of diabetes: estimates for the year 2000 and projections for 2030. Diabetes Care 27, 2568–2569. 15451946 10.2337/diacare.27.10.2568

[2] Lee AJ , Hiscock RJ , Wein P , Walker SP and Permezel M (2007) Gestational diabetes mellitus: clinical predictors and long‐term risk of developing type 2 diabetes a retrospective cohort study using survival analysis. Diabetes Care 30, 878–883. 17392549 10.2337/dc06-1816

[3] Bellamy L , Casas J‐P , Hingorani AD and Williams D (2009) Type 2 diabetes mellitus after gestational diabetes: a systematic review and meta‐analysis. Lancet 373, 1773–1779. 19465232 10.1016/S0140-6736(09)60731-5

[4] Ben‐Haroush A , Yogev Y and Hod M (2004) Epidemiology of gestational diabetes mellitus and its association with type 2 diabetes. Diabet Med 21, 103–113. 14984444 10.1046/j.1464-5491.2003.00985.x

[5] Lappas M , Hiden U , Desoye G , Froehlich J , Mouzon SH‐D and Jawerbaum A (2011) The role of oxidative stress in the pathophysiology of gestational diabetes mellitus. Antioxid Redox Signal 15, 3061–3100. 21675877 10.1089/ars.2010.3765

[6] Karacay O , Sepici‐Dincel A , Karcaaltincaba D , Sahin D , Yalvac S , Akyol M , Kandemir O and Altan N (2010) A quantitative evaluation of total antioxidant status and oxidative stress markers in preeclampsia and gestational diabetic patients in 24–36 weeks of gestation. Diabetes Res Clin Pract 89, 231–238. 20537747 10.1016/j.diabres.2010.04.015

[7] Yu J and Auwerx J (2010) Protein deacetylation by SIRT1: an emerging key post‐translational modification in metabolic regulation. Pharmacol Res 62, 35–41. 20026274 10.1016/j.phrs.2009.12.006 PMC3620551

[8] Kao C‐L , Tai L‐K , Chiou S‐H , Chen Y‐J , Lee K‐H , Chou S‐J , Chang Y‐L , Chang C‐M , Chen S‐J and Ku H‐H (2010) Resveratrol promotes osteogenic differentiation and protects against dexamethasone damage in murine induced pluripotent stem cells. Stem Cells Dev 19, 247–258. 19656070 10.1089/scd.2009.0186

[9] Pardo PS , Mohamed JS , Lopez MA and Boriek AM (2011) Induction of Sirt1 by mechanical stretch of skeletal muscle through the early response factor EGR1 triggers an antioxidative response. J Biol Chem 286, 2559–2566. 20971845 10.1074/jbc.M110.149153 PMC3024751

[10] Zheng Z , Chen H , Li J , Li T , Zheng B , Zheng Y , Jin H , He Y , Gu Q and Xu X (2012) Sirtuin 1 mediated cellular metabolic memory of high glucose via the LKB1/AMPK/ROS pathway and therapeutic effects of metformin. Diabetes 61, 217–228. 22124463 10.2337/db11-0416 PMC3237662

[11] Qiu X , Brown K , Hirschey MD , Verdin E and Chen D (2010) Calorie restriction reduces oxidative stress by SIRT3‐mediated SOD2 activation. Cell Metab 12, 662–667. 21109198 10.1016/j.cmet.2010.11.015

[12] Liu G , Cao M , Xu Y and Li Y (2015) SIRT3 protects endothelial cells from high glucose‐induced cytotoxicity. Int J Clin Exp Pathol 8, 353. 25755722 PMC4348913

[13] Jing E , Emanuelli B , Hirschey MD , Boucher J , Lee KY , Lombard D , Verdin EM and Kahn CR (2011) Sirtuin‐3 (Sirt3) regulates skeletal muscle metabolism and insulin signaling via altered mitochondrial oxidation and reactive oxygen species production. Proc Natl Acad Sci USA 108, 14608–14613. 21873205 10.1073/pnas.1111308108 PMC3167496

[14] Kong X , Wang R , Xue Y , Liu X , Zhang H , Chen Y , Fang F and Chang Y (2010) Sirtuin 3, a new target of PGC‐1α, plays an important role in the suppression of ROS and mitochondrial biogenesis. PLoS One 5, e11707. 20661474 10.1371/journal.pone.0011707 PMC2908542

[15] Caton P , Richardson S , Kieswich J , Bugliani M , Holland M , Marchetti P , Morgan N , Yaqoob M , Holness M and Sugden M (2013) Sirtuin 3 regulates mouse pancreatic beta cell function and is suppressed in pancreatic islets isolated from human type 2 diabetic patients. Diabetologia 56, 1068–1077. 23397292 10.1007/s00125-013-2851-y

[16] Kesavulu M , Giri R , Rao BK and Apparao C (2008) Lipid peroxidation and antioxidant enzyme levels in type 2 diabetics with microvascular complications. Diabetes Metab 26, 387–392. 11119018

[17] Bhutia Y , Ghosh A , Sherpa ML , Pal R and Mohanta PK (2011) Serum malondialdehyde level: surrogate stress marker in the Sikkimese diabetics. J Nat Sci Biol Med 2, 107. 22470243 10.4103/0976-9668.82309 PMC3312689

[18] Panel, I C (2010) International association of diabetes and pregnancy study groups recommendations on the diagnosis and classification of hyperglycemia in pregnancy. Diabetes Care 33, 676–682. 20190296 10.2337/dc09-1848 PMC2827530

[19] Handelsman Y , Mechanick J , Blonde L , Grunberger G , Bloomgarden Z , Bray G , Dagogo‐Jack S , Davidson J , Einhorn D and Ganda O (2011) American Association of Clinical Endocrinologists Medical Guidelines for Clinical Practice for developing a diabetes mellitus comprehensive care plan. Endocr Pract 17(Suppl 2), 1–53. 10.4158/ep.17.s2.1 21474420

[20] Heap J , Murray MA , Miller SC , Jalili T and Moyer‐Mileur LJ (2004) Alterations in bone characteristics associated with glycemic control in adolescents with type 1 diabetes mellitus. J Pediatr 144, 56–62. 14722519 10.1016/j.jpeds.2003.10.066

[21] Brzeszczyńska J , Johns N , Schilb A , Degen S , Degen M , Langen R , Schols A , Glass DJ , Roubenoff R and Greig CA (2016) Loss of oxidative defense and potential blockade of satellite cell maturation in the skeletal muscle of patients with cancer but not in the healthy elderly. Aging (Albany NY) 8, 1690. 27454226 10.18632/aging.101006 PMC5032690

[22] Wang Z , He YL , Cai SR , Zhan WH , Li ZR , Zhu BH , Chen CQ , Ma JP , Chen ZX and Li W (2008) Expression and prognostic impact of PRL‐3 in lymph node metastasis of gastric cancer: its molecular mechanism was investigated using artificial microRNA interference. Int J Cancer 123, 1439–1447. 18561324 10.1002/ijc.23643

[23] Pfaffl MW , Horgan GW and Dempfle L (2002) Relative expression software tool (REST©) for group‐wise comparison and statistical analysis of relative expression results in real‐time PCR. Nucleic Acids Res 30, e36. 11972351 10.1093/nar/30.9.e36 PMC113859

[24] Yuan Y , Cruzat VF , Newshome P , Cheng J , Chen Y and Lu Y (2016) Regulation of SIRT1 in aging: roles in mitochondrial function and biogenesis. Mech Ageing Dev 155, 10–21. 26923269 10.1016/j.mad.2016.02.003

[25] Rodgers JT , Lerin C , Haas W , Gygi SP , Spiegelman BM and Puigserver P (2005) Nutrient control of glucose homeostasis through a complex of PGC‐1α and SIRT1. Nature 434, 113–118. 15744310 10.1038/nature03354

[26] Sun C , Zhang F , Ge X , Yan T , Chen X , Shi X and Zhai Q (2007) SIRT1 improves insulin sensitivity under insulin‐resistant conditions by repressing PTP1B. Cell Metab 6, 307–319. 17908559 10.1016/j.cmet.2007.08.014

[27] Ambra R , Manca S , Palumbo MC , Leoni G , Natarelli L , De Marco A , Consoli A , Pandolfi A and Virgili F (2014) Transcriptome analysis of human primary endothelial cells (HUVEC) from umbilical cords of gestational diabetic mothers reveals candidate sites for an epigenetic modulation of specific gene expression. Genomics 103, 337–348. 24667242 10.1016/j.ygeno.2014.03.003

[28] Balestrieri M , Servillo L , Esposito A , D'Onofrio N , Giovane A , Casale R , Barbieri M , Paolisso P , Rizzo M and Paolisso G (2013) Poor glycaemic control in type 2 diabetes patients reduces endothelial progenitor cell number by influencing SIRT1 signalling via platelet‐activating factor receptor activation. Diabetologia 56, 162–172. 23070058 10.1007/s00125-012-2749-0

[29] Alcendor RR , Gao S , Zhai P , Zablocki D , Holle E , Yu X , Tian B , Wagner T , Vatner SF and Sadoshima J (2007) Sirt1 regulates aging and resistance to oxidative stress in the heart. Circ Res 100, 1512–1521. 17446436 10.1161/01.RES.0000267723.65696.4a

[30] Guan D , Lim J , Peng L , Liu Y , Lam M , Seto E and Kao H‐Y (2014) Deacetylation of the tumor suppressor protein PML regulates hydrogen peroxide‐induced cell death. Cell Death Dis 5, e1340. 25032863 10.1038/cddis.2014.185 PMC4123062

[31] Yang Y , Fu W , Chen J , Olashaw N , Zhang X , Nicosia SV , Bhalla K and Bai W (2007) SIRT1 sumoylation regulates its deacetylase activity and cellular response to genotoxic stress. Nat Cell Biol 9, 1253–1262. 17934453 10.1038/ncb1645 PMC3201724

[32] Schwer B , North BJ , Frye RA , Ott M and Verdin E (2002) The human silent information regulator (Sir) 2 homologue hSIRT3 is a mitochondrial nicotinamide adenine dinucleotide–dependent deacetylase. J Cell Biol 158, 647–657. 12186850 10.1083/jcb.200205057 PMC2174009

[33] Ahn B‐H , Kim H‐S , Song S , Lee IH , Liu J , Vassilopoulos A , Deng C‐X and Finkel T (2008) A role for the mitochondrial deacetylase Sirt3 in regulating energy homeostasis. Proc Natl Acad Sci USA 105, 14447–14452. 18794531 10.1073/pnas.0803790105 PMC2567183

[34] Tao R , Coleman MC , Pennington JD , Ozden O , Park S‐H , Jiang H , Kim H‐S , Flynn CR , Hill S and McDonald WH (2010) Sirt3‐mediated deacetylation of evolutionarily conserved lysine 122 regulates MnSOD activity in response to stress. Mol Cell 40, 893–904. 21172655 10.1016/j.molcel.2010.12.013 PMC3266626

[35] Boyle KE , Newsom SA , Janssen RC , Lappas M and Friedman JE (2013) Skeletal muscle MnSOD, mitochondrial complex II, and SIRT3 enzyme activities are decreased in maternal obesity during human pregnancy and gestational diabetes mellitus. J Clin Endocrinol Metab 98, E1601–E1609. 23956348 10.1210/jc.2013-1943 PMC3790616

[36] Hirschey MD , Shimazu T , Jing E , Grueter CA , Collins AM , Aouizerat B , Stančáková A , Goetzman E , Lam MM and Schwer B (2011) SIRT3 deficiency and mitochondrial protein hyperacetylation accelerate the development of the metabolic syndrome. Mol Cell 44, 177–190. 21856199 10.1016/j.molcel.2011.07.019 PMC3563434

[37] Biri A , Onan A , Devrim E , Babacan F , Kavutcu M and Durak I (2006) Oxidant status in maternal and cord plasma and placental tissue in gestational diabetes. Placenta 27, 327–332. 16338477 10.1016/j.placenta.2005.01.002

[38] Sultan SA , Liu W , Peng Y , Roberts W , Whitelaw D and Graham AM (2015) The role of maternal gestational diabetes in inducing fetal endothelial dysfunction. J Cell Physiol 230, 2695–2705. 25808705 10.1002/jcp.24993

[39] Heerwagen MJ , Miller MR , Barbour LA and Friedman JE (2010) Maternal obesity and fetal metabolic programming: a fertile epigenetic soil. Am J Physiol Regul Integr Comp Physiol 299, R711–R722. 20631295 10.1152/ajpregu.00310.2010 PMC2944425

[40] Strakovsky RS and Pan Y‐X (2012) In utero oxidative stress epigenetically programs antioxidant defense capacity and adulthood diseases. Antioxid Redox Signal 17, 237–253. 22035055 10.1089/ars.2011.4372 PMC6918535

[41] Newsholme P , Cruzat VF , Keane KN , Carlessi R and de Bittencourt PIH (2016) Molecular mechanisms of ROS production and oxidative stress in diabetes. Biochem J 473, 4527–4550. 27941030 10.1042/BCJ20160503C

[42] Newsholme P , Haber E , Hirabara S , Rebelato E , Procopio J , Morgan D , Oliveira‐Emilio H , Carpinelli A and Curi R (2007) Diabetes associated cell stress and dysfunction: role of mitochondrial and non‐mitochondrial ROS production and activity. J Physiol 583, 9–24. 17584843 10.1113/jphysiol.2007.135871 PMC2277225

[43] Loven D , Schedl H , Wilson H , Daabees TT , Stegink LD , Diekus M and Oberley L (1986) Effect of insulin and oral glutathione on glutathione levels and superoxide dismutase activities in organs of rats with streptozocin‐induced diabetes. Diabetes 35, 503–507. 3514329 10.2337/diab.35.5.503

[44] Wohaieb SA and Godin DV (1987) Alterations in free radical tissue‐defense mechanisms in streptozocin‐induced diabetes in rat: effects of insulin treatment. Diabetes 36, 1014–1018. 3301471 10.2337/diab.36.9.1014

[45] Gawlik K , Naskalski J , Fedak D , Pawlica‐Gosiewska D , Grudzień U , Dumnicka P , Małecki M and Solnica B (2015) Markers of antioxidant defense in patients with type 2 diabetes. Oxid Med Cell Longev 2016, 2352361. 10.1155/2016/2352361 PMC4657103 26640613

[46] Roca‐Rodríguez M , López‐Tinoco C , Murri M , Fernández‐Deudero A , Garcia‐Palacios M , Garcia‐Valero M , Tinahones‐Madueno F and Aguilar‐Diosdado M (2014) Postpartum development of endothelial dysfunction and oxidative stress markers in women with previous gestational diabetes mellitus. J Endocrinol Invest 37, 503–509. 24458829 10.1007/s40618-013-0045-6

